# A Prospective, Randomized Study Comparing the Outcome After Thulium Laser Enucleation of the Prostate with Conventional Monopolar TURP for the Treatment of Symptomatic Benign Prostatic Hyperplasia

**DOI:** 10.5152/tud.2024.23128

**Published:** 2024-01-01

**Authors:** Ajaykumar Dhirubhai Tadha, Debansu Sarkar, Dilip Kumar Pal

**Affiliations:** Department of Urology, Institute of Post Graduate Medical Education and Research & SSKM Hospital, Kolkata, West Bengal, India

**Keywords:** BPH, ThuLEP, TURP

## Abstract

**Objective:**

This is a prospective randomized study with the aim of comparing (thulium laser enucleation of the prostate (ThuLEP) and transurethral resection of the prostate (TURP) for benign prostatic hyperplasia (BPH) treatment.

**Methods:**

Patients are assessed preoperatively and up to 6 months postoperatively. International Prostate Symptom Score (IPSS), quality of life (QoL), maximum urinary flow rates (Qmax), international index of erectile function-5 (IIEF-5) and post-void residual volume (PVR) are collected on each follow-up.

**Results:**

In comparison to TURP, the ThuLEP group has significantly less need for catheter traction and less need for postoperative irrigation. The operative time is significantly higher in ThuLEP compared to TURP. ThuLEP is significantly superior to TURP in terms of early catheter removal, less drop in haemoglobin, less fall in serum sodium level, and early hospital discharge. ThuLEP and TURP resulted in a significant improvement from baseline in terms of IPSS, PVR, Qmax, and QoL, but there was no significant difference between the 2 groups. The IIEF-5 is the same as the baseline in both groups. Early and late complications are also comparable.

**Conclusion:**

The ThuLEP outperforms TURP in terms of blood loss, significantly less need for postoperative catheter traction, bladder irrigations, early catheter removal, and less hospital stay. Transurethral resection of the prostate takes longer operative time in the early stages of experience. The results of both surgeries are comparable in terms of PVR, Qmax, and subjective scoring systems (IPSS, QoL). Transurethral resection of the prostate is a safe and efficient BPH treatment method comparable to the monopolar TURP.

**Main Points**

- In compared to m-TURP, ThuLEP has longer operative time.
- ThuLEP has favorable perioperative course in terms of less intraoperative blood loss, less need of catheter traction and early catheter removal.
- The short term Outcome are comparable in both ThuLEP and m-TURP.

## Introduction

Bladder outlet obstruction (BOO) secondary to benign prostatic hyperplasia (BPH) is a common situation an urologist finds in his day-to-day practice. Although it is simple to make a diagnosis of benign prostatic obstruction in OPD, the operative treatment of the condition is complex and still evolving. The evolution of prostate surgery for BPH started with open prostatectomy. With time and the advent of endourological treatment, monopolar transurethral resection of the prostate (TURP) became the gold standard treatment, but this was associated with complications such as blood loss, excessive fluid absorption, erectile dysfunction, incontinence, and TUR (Transurethral resection) syndrome.^1^ It is also a prostate-volume-bound surgery.^1^ To overcome this barrier, various lasers were applied for BPH. The technique of surgery also went through modifications, from chip removal to incision, evaporation, and finally enucleation of the whole prostate.^2^ Neodymium-doped yttrium aluminum garnet (Nd: YAG), diode, potassium-titanyl-phosphate (KTP), holmium, and more recently, thulium laser, all are applied for treatment on prostatic tissue.^3^

In recent literature, holmium laser enucleation (HoLEP) has proven its efficacy and safety in managing BPH, especially since it is suitable for patients with larger prostate volumes and who are on anticoagulants.^4,5^ Thulium is the new laser that was introduced in clinical practice in 2005 with a wavelength between 1.75 mm and 2.22 mm.^6^ In comparison to the holmium laser, this laser has several advantages that include more precise tissue incision, improved spatial beam quality, and the ability to operate in both continuous and pulsed modes.^6^ At this wavelength, laser absorption is higher, leading to more efficient and rapid tissue cutting.^6^ The thulium laser is proven suitable for bladder neck incision, transurethral vaporization, or vapo-resection, and recently in thulium laser enucleation of the prostate (ThuLEP) in various literature.^7^

After the description of the procedure by Herrmann et al,^2^ various investigators have published studies related to and in favor of ThuLEP. When we search for a comparison between ThuLEP and standard monopolar TRUP, the literature is very scant. Hence, we designed this study to compare both procedures at our institute.

### Material and Methods

This is a randomized prospective study conducted at a tertiary care hospital in India (Institute of Post-Graduate Medical Education and Research Center, Kolkata). Institutional ethical committee approval was obtained from Institute of Post-Graduate Medical Education and Research Center before the commencement of the study (Approval No: IPGM E&R/IEC/20 21/31 3). The study period was from February 2021 to September 2022. The inclusion criteria of the study were male patients with an age between 50 and 85 years with bothersome LUTS (lower urinary tract symptoms), failed drug therapy, a maximum urinary flow rate (Qmax) < 15 mL/s, recurrent hematuria of prostatic pathology, refractory urinary obstruction, recurrent urinary tract infections, bladder diverticula secondary to BOO, and obstructive uropathy secondary to BOO. The exclusion criteria of our study were neurogenic bladder, diagnosed prostatic cancer, previous prostatic, bladder-neck, or urethral surgery, a patient not giving consent for surgery, and a patient who had cardiopulmonary compromise and other conditions rendering them unfit for prostatic surgery.

Randomization and data collection forms: After institutional ethical committee clearance, patients are randomized into 2 groups by a lottery system. A proforma containing various parameters under study is used for data collection. Data collection started after obtaining the informed consent of the patient.

Statistical Analysis

Thesample size has been calculated with the help of Epi Info^™^ 3.5.3. The sample size needed for this study is 35 patients in both groups. Statistical analysis is done using a Microsoft Excel spreadsheet and analyzed by Statistical Package for the Social Sciences Statistics software, version 27.0 (IBM SPSS Corp.; Armonk, NY, USA), and GraphPad Prism (version 5). The data has been summarized as the mean and standard deviation for numerical variables and the count and percentages for categorical variables. Unpaired proportions were compared by the Chi-square test or Fisher’s exact test, as appropriate. Two sample *t*-tests for a mean difference involved independent or unpaired samples. *P*-value ≤ .05 is considered statistically significant.

The specification of the thulium laser: We used a thulium laser from Cyber^TM^ (Quanta System) with an optical power output of 150 W. Enucleation was performed in pulsed laser mode at 60 W, and coagulation setting was set at 50 W.

The comparison is made on the following points: 1) intraoperative: operative time, total weight of tissue resected; 2) postoperative period: analgesics requirements, the requirement of catheter traction, drop in hemoglobin, blood transfusion rate, change in serum sodium level, rate of occurrence of TUR syndrome, postoperative catheterization time, post-void residual (PVR) volume, uroflowmetry, and postoperative hospital stay; 3) follow-up: International Prostate Symptoms Score (IPSS) and quality of life (QoL) score improvement, change in International Index of Erectile Function (IIEF) score, incontinence episode, post-void residual urine volume, uroflowmetry, and surgical complications in the postoperative period.

Study method and protocol of follow-up: Patients admitted with LUTS due to BPH were evaluated by scoring subjective symptoms with the help of the IPSS, QoLs, and IIEF questionnaires; physical examination and digital rectal examination (DRE) were performed; measurement of total serum prostate-specific antigen (PSA); USG KUB; and TRUS (trans-rectal ultrasound) measurement of prostate volume; PVR volume, and Qmax on uroflowmetry. In patients with suspected age-specific PSA values or suspected DRE, a TRUS-guided needle biopsy of the prostate is done. Investigations like a complete blood count and renal function test with measurement of serum electrolytes are done in each patient.

Fluoroquinolone, or third-generation cephalosporin, was given to each patient 30 minutes before the surgery. Intraoperative parameters were noted as per pro forma including the weight of the prostatic tissue specimen extracted. Catheter traction was applied in selected cases where there was mild hematuria after catheter insertion. Catheter traction was applied on the leg and was released once the hematuria subsided. Irrigation was applied to each case and continued for 6 hours after hematuria resolved.

Postoperatively, each patient was given round-the-clock intravenous analgesics for a period of the first 24 hours. After this period, the analgesics were given only on requirement. A complete blood count and serum sodium level were checked within 6 hours of the surgery. The patient was closely assessed for symptoms of TUR syndrome postoperatively. A fall in hemoglobin by >1 g was considered significant. A postoperative blood transfusion was performed only if the hemoglobin was <8 g after the surgery. Each patient was assessed for the feasibility of catheter removal on day 1. A trial void without a catheter was given only when the urine was clear. After catheter removal, the post-void residual urine and uroflowmetry were recorded. The patient was discharged after a normal void following catheter removal.

Each patient was followed up on days 10, 30, 3 months, and 6-month period. Qmax and PVR were assessed on each visit. IPSS, QoLs, and IIEF questionnaires were noted in the second, third, and fourth visits. Kidney–ureter–bladder ultrasound (USG KUB) was performed on the fourth visit. Patients were assessed for surgical complications like incontinence, bladder neck stenosis, or urethral stricture disease if the IPSS/QoLs questionnaire suggested so.

## Results

Thirty-five patients were included based on inclusion and exclusion criteria in both groups and operated accordingly. The collected data are analyzed. Various causes for which the surgery was performed are mentioned in Table 1. Both groups are comparable in terms of patient age, serum PSA, preoperative serum creatinine, and prostate volume. Intraoperative time is significantly higher in the ThuLEP group and the weight of prostate tissue resected is significantly lower in the ThuLEP group, compared to the other groups. In the TURP group, 2 patients required blood transfusions, and none were required in the ThuLEP group. There is a significant drop in serum sodium levels in the TURP group, but none developed clinically significant hyponatremia.

Table 2 shows some of the post-operative comparisons. The ThuLEP group had significantly less need for analgesia and blood transfusion. Traction was not needed in 2 patients in TURP group and 9 patients in ThuLEP group. Traction was removed (if applied) once the urine is clear. The irrigation time, total catheter time, and hospital stay was significantly less with ThuLEP. Post-operative data analysis showed significant improvement in each parameter (IPSS, QoLs, Qmax, and PVR) from baseline in both groups except IIEF (Table 3).

## Discussion

The TURP has been the gold standard procedure for BPH for decades.^8^ Gilling (in 1988) was the first to describe enucleation by HoLEP for treating BPH and showed good results.^4^ In recent years, with the increased availability of the holmium laser, HoLEP has been considered the best procedure for treating prostate enlargement.^5^ The thulium laser was introduced for clinical use in 2005 and has the advantages of smaller size, precise tissue incision, and more efficient operation compared to the holmium laser.^6^ Hermann et al^2^ (2010) published the first article mentioning the ThuLEP procedure in detail, which is believed to achieve maximum surgical results and minimal side effects regardless of prostate size. In recent years, comparisons between bipolar TURP and HoLEP have been done in various studies.^5^ A plethora of literature is also available on the comparison of HoLEP and ThuLEP. When we search for evidence of ThuLEP compared to the present gold standard TURP, data are very limited. Most of this literature compares ThuLEP with bipolar TURP.

After Hermann’s description of ThuLEP, the only study comparing standard m-TURP to ThuLEP was published by Swiniarski et al (2012).^9^ They randomized patients into 2 groups: 54 patients in the ThuLEP group and 52 patients in the TURP group, followed up for 3 months. Perioperative parameters were noted. Before and after surgery, IPSS, QoLs, Qmax, and PVR were noted. They found that ThuLEP surgery was associated with less blood loss and more operative time. Commenting on the longer operative time in ThuLEP, the authors have pointed out 2 issues. First of all is the experience of the surgeon; ThuLEP was the newer technique, and surgeons were in different parts of their experience, but TURP was well established and had almost comparable skill among surgeons. Secondly, the individual predispositions and preferences of the operating surgeon determine the speed and manner of performing the surgery. The weight of tissue resected was also significantly less in the ThuLEP group. Tissue vaporization associated with the thulium laser could explain differences concerning the weight of the resected tissue. They also noted that short-term outcomes like IPSS, QoLs, Qmax, and PVR were improved in both groups, but the results are comparable. Both groups were comparable in terms of complication rates. With these findings, they concluded ThuLEP was a safe and efficient treatment for BPH with comparable outcomes to TRUP in a 3-month observation.

In our study, ThuLEP surgery took a significantly longer time than TURP. The reason is an early part of the learning process for the surgeon, and the secondary motive of the surgery was to demonstrate the procedure for educational purposes. Intraoperative hemostasis was good in the ThuLEP group as compared to m-TURP. This can be statistically indicated by a less drop in hemoglobin level. The ThuLEP group rarely required coagulation on the prostate bed once the enucleation is done, while in TURP, there were always some bleeders that needed to be found and coagulated before proceeding further. We also noticed a significant fall in serum sodium levels after both ThuLEP and TURP groups. The fall was more significant in the TURP group. We did not expect a fall in sodium level in the former procedure but none of our patients develop clinical symptoms of hyponatremia.

Thulium laser enucleation of the prostate is also known for its effect on tissue vaporization.^10^ In various studies, it is noted that around 20 to 30% of the tissue is vaporized during the process of enucleation.^8,11^ In our study, the weight of tissue collected after ThuLEP was significantly less than the weight of tissue resected after TURP surgery. The tissue after ThuLEP is very small and may get stuck in the net placed for collecting the specimen. This was the one practice issue we faced during collecting tissue and may be one of the reasons for less tissue retrieval in ThuLEP. These findings are in line with the above-mentioned study.^9^

Thulium laser enucleation of the prostate showed the main advantage in the postoperative recovery period. Traction was applied in all initial cases of ThuLEP, even after good hemostasis, but as we gained more confidence, we started traction-free ThuLEP (n = 9). In the TURP group, all except 2 required catheter traction. Traction, if applied, was removed once the effluent was clear of any blood. The mean traction time is significantly less in the ThuLEP group. Irrigation was continued until 6 hours after the hematuria subsided. With this, the mean time of irrigation and the mean requirement of analgesics were significantly less in the ThuLEP group. The time needed for catheter removal and hospital stay is also less in ThuLEP, which is in line with findings published by other investigators as well.^9,11^

On follow-up day -30, IPSS, QoLs, Qmax, and PVR were improved in both groups, but the difference is non-significant. This improvement is maintained for up to 6-month follow-ups in both groups. The IIEF score remained unaffected in both groups at 6 months. Bladder mucosal injury occurred in one initial case during the morcellation process, which was managed conservatively. This incident taught us the importance of keeping the bladder distended during the process of morcellation. One patient in ThuLEP developed postoperative urethritis. Six patients in ThuLEP and 7 patients in the TURP group had incontinence episodes, but all were temporary and improved with time. One patient in the TURP group developed urosepsis, which was managed with antibiotics. We did not face any long-term complications in either group. The complications in both groups are statistically comparable. Similar findings were published by Swiniarski et al (2012).^9^

Transurethral resection of the prostate is considered a prostate volume-bound surgery.^1^ It has been observed in previous studies that as the weight of tissue increases, the complication rate also increases after TURP. In 2019, Chang et al published their experience of using a thulium laser for enucleation of >80 mL of gland.^12^ They used a 150-200 W thulium laser on 336 patients and found thuLEP is safe and effective for patients with >80 mL prostate glands. They concluded that high-power ThuLEP is feasible for patients who are otherwise not candidates for endoscopic treatment. This suggests that ThuLEP is a size-independent procedure, unlike TURP. In our study, the mean prostate volume in both groups was around 70 g. Seven patients in the ThuLEP group and 6 patients in the TURP group had prostate sizes >80 g, and in all patients, the prostate resection was satisfactory.

Yang et al (2013)^11^ and Bozzini et al (2017)^13^ showed ThuLEP as a safe procedure and superior to bipolar TURP with comparable complications. A meta-analysis published by Chen et al (2020) concluded that ThuLEP is a superior procedure to TURP and HoLEP.^14^ A few other investigators have also concluded that ThuLEP is a superior enucleation technique to HoLEP.^8,15,16^

In comparison to monopolar TURP, ThuLEP is associated with a favorable perioperative period, except for the longer operative time. ThuLEP showed similar improvement in postoperative parameters (IPSS, QoL score, Qmax, IIEF, and PVR) as well as a similar rate of short-term complications compared to the TURP group. These led us to conclude that ThuLEP is a safe and effective procedure for the treatment of BPH, and our results are comparable to the present gold standard, TURP. This improvement was maintained after 6 months of follow-up in both groups.

## Figures and Tables

**Table 1.** Various Causes of Benign Prostatic Hyperplasia Surgery in Both Groups

Indication of Surgery	ThuLEP	TURP
Refractory urinary retention	17	15
Obstructive uropathy	9	8
Bothersome LUTS	9	11
Recurrent UTI	0	1
Total	35	35

LUTS, lower urinary tract symptoms; ThuLEP, thulium laser enucleation of the prostate; TURP, transurethral resection of the prostate; UTI, urinary tract infection.

**Table 2.** Baseline Parameters, Preoperative, Intraoperative, and Postoperative Parameter Comparison Between Thulium Laser Enucleation of the Prostate and Transurethral Resection of the Prostate Groups

		ThuLEP	TURP	*P* (ThuLEP vs. TURP)
Number of Patients		35	35	
Baseline parameters	Age (years)	65.51 ± 8.56	65.60 ± 8.25	.483
	No. of patients without catheter	9	12	3
	No. of patients with catheter	26	23	
Preoperative parameter	S.PSA (ng/mL)	3.14 ± 1.49	3.55±1.5	.128
	Pre-op creatinine (mg/dL)	1.38 ± 0.69	1.63 ± 1.19	.148
	Prostrate weight (g)	69.74 ± 15.3	70.09 ± 13.59	**.461**3
Intraoperative parameter 3	Operative time (min)	84.80 ± 22.59	70.86 ± 14.44	**.002**3
	Weight of tissue resected (g)	45.80 ± 11.35	51.31 ± 12.61	**.029**3
Postoperative parameters	Drop in serum sodium level (meq/dL)	3.03 ± 1.38	6.51 ± 2.06	**<.001**3
	Analgesics required (h)	8.00 ± 2.74	10.23 ± 2.1	**<.001**3
	Traction Status (h)	7.94 ± 5.63	12.63 ± 3.52	**<.001**3
	Irrigation Time (h)	14.91 ± 7.51	20.49 ± 5.01	**<.001**3
	Drop in hemoglobin (g %)	0.82 ± 0.5	1 ± 0.56	**<.001**3
	Blood transfusion—Yes	0%	2(5.71%)	**.076**3
	Blood transfusion—No	35 (100.00%)	33 (94.29%)	–
	Time of catheter removal (h)	29.80 ± 6.4	38.25 ± 8.45	**<.001**3
	PVR after catheter removal (mL)	22.20 ± 6.69	19.78 ± 6.23	**.073**3
	Hospital stay (h)	43.14 ± 8.31	50.40 ± 7.7	**<.001**3

PVR, post-void residual; ThuLEP, thulium laser enucleation of the prostate; TURP, transurethral resection of the prostate.

**Table 3.** Comparison of baseline International Prostate Scoring System, Maximal Flow Rate, Quality of Life Scores, International Index of Erectile Function, and Post-void Residual of Thulium Laser Enucleation of the Prostate and Transurethral Resection of the Prostate group to the same parameters on follow-up day 10, day 30, day 90, and day 180

			Preop.	Day 10 (*P*-Value in Comparison to Pre-op)	Day 30 (*P*-Value in Comparison to Pre-op)	Day 90 (*P*-Value in Comparison to Pre-op)	Day 180 (*P*-Value in Comparison to Pre-op)
IPSS	ThuLEP	Mean ± SD	23.06 ± 4.22	NR	3.03 ± 1.4 (<.001)	2.09 ± 1.5 (<.001)	2.17 ± 1.15 (<.001)
	TURP	Mean ± SD	24.09 ± 2.36	NR	3.77 ± 1 (<.001)	2.37 ± 0.88 (<.001)	2.23 ± 0.73 (<.001)
	*P* (ThuLEP vs. TURP)		.106	-	.607	.167	.402
Qmax	ThuLEP	Mean ± SD	6.53 ± 1.63	20.88 ± 3.62 (<.001)	24.67 ± 2.26 (<.001)	27.29 ± 1.75 (<.001)	28.00 ± 1.71 (<.001)
	TURP	Mean ± SD	6.10 ± 1.63	16.80 ± 2.56 (<.001)	22.06 ± 2.05 (<.001)	25.67 ± 1.44 (<.001)	26.80 ± 1.34(<.001)
	*P* (ThuLEP vs. TURP)		.064	.090	.121	.150	.120
QoLs	ThuLEP	Mean ± SD	5.06 ± 0.68	NR	0.31 ± 0.47 (<.001)	0.23 ± 0.6 (<.001)	0.34 ± 0.54 (<.001)
	TURP	Mean ± SD	5.03 ± 0.71	NR	0.29 ± 0.46 (<.001)	0.34 ± 0.48 (<.001)	0.29 ± 0.46 (<.001)
	*P* (ThuLEP vs. TURP)		**.432**3	**-**3	**.399**3	**.191**3	**.317**3
IIEF	ThuLEP	Mean ± SD	19.34 ± 4.7	NR	19.46 ± 4.55 (0.385)	19.38 ± 3.64 (0.475)	19.45 ± 3.64 (0.475)
	TURP	Mean ± SD	17.94 ± 3.23	NR	18.51 ± 2.49 (0.021)	18.46 ± 2.83 (0.059)	18.23 ± 2.73 (0.051)
	*P* (ThuLEP vs. TURP)		**.075**3	**-**3	**.143**3	**.130**3	**.100**3
PVR	ThuLEP	Mean ± SD	121.09 ± 63.45	20.06 ± 9.62 (<.001)	14.86 ± 7.94 (<.001)	11.74 ± 7.94 (<.001)	9.80 ± 7.94 (<.001)
	TURP	Mean ± SD	110.14 ± 62.82	17.06 ± 5.01 (<.001)	13.57 ± 5.76 (<.001)	9.49 ± 6.32 (<.001)	8.10 ± 6.32 (<.001)
	*P* (ThuLEP vs. TURP)		**.235**3	**.054**3	**.219**3	**.096**3	**.096**3

IIEF, International Index of Erectile Function; IPSS, International Prostate Scoring System; NR, not recorded; PVR, post-void residual; Qmax, maximal flow rate; QoLs, quality of life scores; ThuLEP, thulium laser enucleation of the prostate; TURP, transurethral resection of the prostate.
